# The impact of post-radioiodine therapy SPECT/CT on early risk stratification in differentiated thyroid cancer; a bi-institutional study

**DOI:** 10.18632/oncotarget.19781

**Published:** 2017-08-01

**Authors:** Szabina Szujo, Livia Sira, Laszlo Bajnok, Beata Bodis, Ferenc Gyory, Orsolya Nemes, Karoly Rucz, Peter Kenyeres, Zsuzsanna Valkusz, Krisztian Sepp, Erzsebet Schmidt, Zsuszanna Szabo, Sarolta Szekeres, Katalin Zambo, Sandor Barna, Endre V. Nagy, Emese Mezosi

**Affiliations:** ^1^ Ist Department of Medicine, University of Pecs, H-7624 Pecs, Hungary; ^2^ Department of Medicine, Faculty of Medicine, University of Debrecen, H-4012 Debrecen, Hungary; ^3^ Department of Nuclear Medicine, University of Pecs, H-7624 Pecs, Hungary; ^4^ Department of Nuclear Medicine, Faculty of Medicine, University of Debrecen, H-4012 Debrecen, Hungary; ^5^ Department of Surgery, Faculty of Medicine, University of Debrecen, H-4032 Debrecen, Hungary; ^6^ Ist Department of Medicine, University of Szeged, H-6720 Szeged, Hungary

**Keywords:** differentiated thyroid cancer, radioiodine therapy, SPECT/CT, ATA risk classification, ETA risk classification

## Abstract

**Objective:**

SPECT/CT has numerous advantages over planar and traditional SPECT images. The aim of this study was to evaluate the role of post-radioiodine therapy SPECT/CT of patients with differentiated thyroid cancer (DTC) in early risk classification and in prediction of late prognosis.

**Patients and methods:**

323 consecutive patients were investigated after their first radioiodine treatment (1100–3700 MBq). Both whole body scan and SPECT/CT images of the head, neck, chest and abdomen regions were taken 4–6 days after radioiodine therapy. Patients were re-evaluated 9–12 months later as well as at the end of follow up (median 37 months).

**Results:**

Post-radioiodine therapy SPECT/CT showed metastases in 22% of patients. Lymph node, lung and bone metastases were detected in 61, 13 and 5 patients, respectively, resulting in early reclassification of 115 cases (36%). No evidence of disease was found in 251 cases at 9–12 months after radioiodine treatment and 269 patients at the end of follow-up. To predict residual disease at the end of follow-up, the sensitivities, specificities and diagnostic accuracies of the current risk classification systems and SPECT/CT were: ATA: 77%, 47% and 53%; ETA: 70%, 62% and 64%; SPECT/CT: 61%, 88% and 83%, respectively. There was no difference between cohorts of the two institutions when data were analyzed separately.

**Conclusions:**

Based on our bi-institutional experience, the accuracy of post-radioiodine SPECT/CT outweighs that of the currently used ATA and ETA risk classification systems in the prediction of long-term outcome of DTC.

## INTRODUCTION

The worldwide incidence of thyroid cancer has continuously increased during the last few decades. This rise can be attributed to the increased diagnosis of occult cancers through the use of neck ultrasound and other techniques of diagnostic neck imaging [1–5]. In Hungary, approximately 800 new cases were registered by the National Cancer Registry and Biostatistics Center in 2014 (http://www.onkol.hu/hu/nemzeti_rakregiszter, accessed in February 07, 2017). Although the use of improved techniques leads to earlier and more accurate diagnosis, it may result in overdiagnosis and overtreatment; there is an urgent need to better distinguish the high-risk patients requiring therapy from those who do not need radioiodine after surgery, or may not need treatment at al. [3].

Patients with differentiated thyroid cancer (DTC) usually have a favorable prognosis with high cure rates; however, lifelong follow-up is required as potentially curable local recurrences and distant metastases may occur even decades later [6–8]. The conventional and effective treatment consists of surgical management followed by radioiodine (RAI) ablation of thyroid remnants and thyroid-stimulating hormone (TSH) suppressive therapy [6, 7]. Recently, the universal use of remnant ablation after surgery has been debated and mainly restricted to advanced disease [8]. However, radioiodine therapy has additional benefits, e.g. the destruction of undetected residual tumor foci and the ablation of normal thyroid tissue which facilitates the detection of recurrent disease during follow up. The information obtained through the posttherapeutic ^131^I whole-body scan (WBS) or single photon emission computed tomography/computed tomography (SPECT/CT) may reveal previously undiagnosed tumor foci [9–12]. Planar WBS is routinely performed after radioiodine treatment; however, SPECT/CT is proven to be more accurate in the evaluation of residual disease [13–16]. Hybrid systems − integrating a SPECT camera with a computed tomography (CT) scanner in one gantry − have been in use since 2001, and in the last 5 years the application of SPECT/CT imaging system is gaining more importance [17]. In comparison to WBS, SPECT/CT adds simultaneous 3D anatomic mapping to functional imaging [18]. The recognition of artifacts is easier and metastatic foci without radioiodine uptake are also detected [19]. SPECT/CT significantly ameliorates the diagnosis and staging, and differentiates between benign and malignant foci of radioiodine accumulation [20].

Postoperative and follow-up management of patients with DTC highly depends on risk classification. Different risk stratification systems are used by the American Thyroid Association (ATA, 2009, 2015) and the European Thyroid Association (ETA, 2006). Risk stratification systems incorporate data from cancer related factors, clinical features, results of first WBS after radioiodine therapy and serum thyroglobulin (Tg) level. The evaluation of response to initial therapy during the follow-up is especially important; risk categories may change during the course of disease. The reclassification of patients based on post-radioiodine therapy imaging influences the management of the disease and the intensity of follow-up [21].

The aim of this study was to evaluate the clinical utility of combined imaging with SPECT/CT after radioiodine treatment of patients with DTC in early risk classification and in prediction of late prognosis. These results have been compared to the currently used ATA and ETA risk stratification systems.

## MATERIALS AND METHODS

### Characteristics of the patients

After their first radioiodine treatment, 323 consecutive DTC patients (181 at the University of Pecs and 142 at the University of Debrecen) were investigated. Demographic data are summarized in Table 1. The female to male ratio was 246 to 77. The median age at diagnosis was 46 (range 13 to 86) years. All patients were diagnosed with DTC; papillary and follicular histotypes were identified in 249 and 74 cases, respectively. Among the papillary tumors, classical papillary variant was the most common subtype (75.1%), while the incidence rate of follicular (20.9%), sclerosing (2.4%) and tall cell variants (1.6%) was lower. Of the follicular tumors, classical variant was the most common (81.1%); Hürthle-cell, trabecular and insular variants were diagnosed in 16.2% 1.3% and 1.3% of cases, respectively. Histology detected lymph node involvement in 95 cases, distant metastases were known in 12 patients. Thyroglobulin antibody (TgAb) positivity was found in 88 patients.

**Table 1:** Patients’ demographics (*n* = 323)

Characteristics	*n* (%)
*Age (years)* Median (range)	46 (13–86)
*Gender* Female Male	246 (76.2) 77 (23.8)
*Tumor histology* *Papillary (PTC)* Classical Follicular Sclerosing Tall cell *Follicular (FTC)* Classical Hürthle cell Trabecular Insular	249 (77.1) 187 (75.1) 52 (20.9) 6 (2.4) 4 (1.6) 74 (22.9) 60 (81.1) 12 (16.2) 1 (1.3) 1 (1.3)
*T stage* T1 T2 T3 T4 *N stage* N0 N1 *M stage* M0 M1 *pTNM staging* I II III IV	143 (44.3) 79 (24.5) 78 (24.3) 21 (6.5) 228 (70.6) 95 (29.4) 311 (96.3) 12 (3.7) 219 (67.8) 28 (8.7) 36 (11.1) 40 (12.4)
*TgAb* Negative Positive	235 (72.8) 88 (27.2)

The study protocol was approved by the Institutional Ethics Committees of the University of Pecs and the University of Debrecen.

### RAI ablation

Based on the surgical and pathological status of the tumor, patients were classified into risk groups according to the European consensus guideline [7]. Patients with low risk for recurrence, younger than 45 years and without aggressive histology were treated with 1100 MBq, while other patients received 3700 MBq doses. In order to reach effective thyroid ablation, two methods of preparation were available: thyroid hormone withdrawal or administration of recombinant human thyrotropin (rhTSH, 34 patients). Total body retention and external radiation dose were measured before discharge of the patient.

### Post-radioiodine therapy imaging with WBS and SPECT/CT

Both planar whole body scan (WBS) and SPECT/CT from the neck and chest were carried out in all patients 4–6 days after oral administration of 1100–3700 MBq radioiodine. Additional SPECT/CT scans of the abdomen and pelvis were acquired if suspicious isotope accumulations were detected on the WBS. The WBS examination consisted of anterior and posterior whole-body images acquired at 6 cm/min using a DHV SPECT/CT equipment (Mediso, Budapest, Hungary). The SPECT/CT unit consisted of dual head SPECT, 50 sec/frame, 64 frames, and a low dose, 16 slices spiral CT, 120 KeV, 50 mAs [22]. The examination was carried out with HEGP collimator. Evaluation of WBS and SPECT/CT images were performed by two independent nuclear medicine specialists and a radiologist; in case of dissent opinion, consensus was achieved. The CERTOP 01-13044/6/11-07755 Quality Management System was applied, which satisfied the requirements of the Hungarian Health Care Standards Guide (MEES) 1.0. Identical protocols were used at both university centers.

### Risk classification systems and SPECT/CT based upgrading and downgrading rules

The risks of recurrence were calculated separately according to both the ATA 2009 and ETA 2006 guidelines.

According to the ATA risk stratification system, patients can be categorized as (1) low risk, if there are no local or distant metastases, all macroscopic tumor has been removed, no tumor invasion of local regional tissues, no aggressive histology or vascular invasion, no RAI uptake outside the thyroid bed on the first posttreatment WBS are present; (2) intermediate risk, if there are microscopic invasion of the tumor into the perithyroidal tissue, or cervical lymph node metastases are present, or there is RAI uptake outside the thyroid bed on the first posttreatment WBS, or aggressive histology or vascular invasion, and (3) high risk, if there is macroscopic tumor invasion, or incomplete tumor resection, or distant metastases, or thyroglobulinemia out of proportion to what is seen on the posttreatment scan [6].

According to the ETA risk classification the risk is (1) very low if the tumor is unifocal T1 (≤ 1 cm) N0M0 and there is no extension beyond the thyroid capsule; (2) low, if the tumor is T1 (> 1 cm) N0M0, or T2N0M0, or multifocal T1N0M0; (3) high if the tumor is any T3; any T4; any T with N1 or M1 [7].

The risk of recurrence was reevaluated based on SPECT/CT results. Patients without RAI uptake outside the thyroid bed, except those with aggressive histology, were downgraded, while those with detected tumor foci were upgraded according to the ATA classification.

The SPECT/CT results served as a basis for separating patients into two groups: those with or without residual tumor.

Serum Tg, TgAb, neck ultrasound and other imaging modalities were used during reclassification of patients at 9–12 months after the RAI treatment. No evidence of tumor was established with negative neck ultrasound, on-thyroxine Tg < 0.2 ng/ml or stimulated Tg < 2.0 ng/ml, negative TgAb or significant decrease in TgAb titer. Incomplete biochemical response was determined if Tg was measurable or TgAb titer did not decrease without morphological abnormality. Structural disease was diagnosed with positive imaging findings.

### TSH, Tg and TgAb measurements

Thyroid stimulating hormone (TSH) was measured by an electrochemiluminescence assay (Elecsys^®^ TSH assay, Roche, measuring range: 0.005–100 µIU/mL).

For the University of Pecs patients, Tg and TgAb were measured by Elecsys^®^ TG II assay (Roche, measuring range of 0.04–500 ng/mL) and Elecsys^®^ anti-TG assay (Roche, measuring range of 10.0–4000 IU/ml), respectively.

For the University of Debrecen patients, Tg was measured by chemiluminescent immunoassay (LIAISON^®^-Tg, DiaSorin S.p.A., Saluggia, Italy; measuring range: 0.2–1000 ng/ml). Before December 1, 2014 concentrations of TgAb were measured by radioimmunoassay (DYNOtest anti-Tg, BRAHMS Diagnostica GmbH, Hennigsdorf, Germany; measuring range: 20–2000 U/ml); from December 1, 2014 the Elecsys^®^ anti-TG assay (Roche, measuring range: 10.0–4000 IU/ml) was used.

### Data analysis

Statistical analysis was done with Statistical Package for the Social Sciences (SPSS, Inc., Chicago, IL, USA, version 22.0). Normality of distribution of data was tested by Kolmogorov-Smirnov test. Non-normally distributed parameters were presented as median and ranges. The diagnostic value of risk classification systems to predict the recurrence of tumor at one-year and at the end of follow-up was calculated according to Galen. It was based on true-positive (TP), true-negative (TN), false-positive (FP), and false-negative (FN) results: sensitivity: TP/TP+FN, specificity: TN/TN+FP, positive predictive value (PPV): TP/TP+FP, negative predictive value (NPV): TN/(TN+FN) and diagnostic accuracy: (TP+TN)/(TP+TN+FP+FN). The agreement between risk stratification systems was calculated with Cohen’s *kappa* coefficient. The diagnostic value of different risk classification systems were compared by McNemar test. For comparison to other systems, ATA intermediate and high risk categories were handled together. To identify the determinants of disease outcome, binary logistic regression analysis using backward method was performed. We considered *p* < 0.05 to be significant for all analyses.

## RESULTS

### SPECT/CT after the first ^131^I treatment

**Figure 1:**
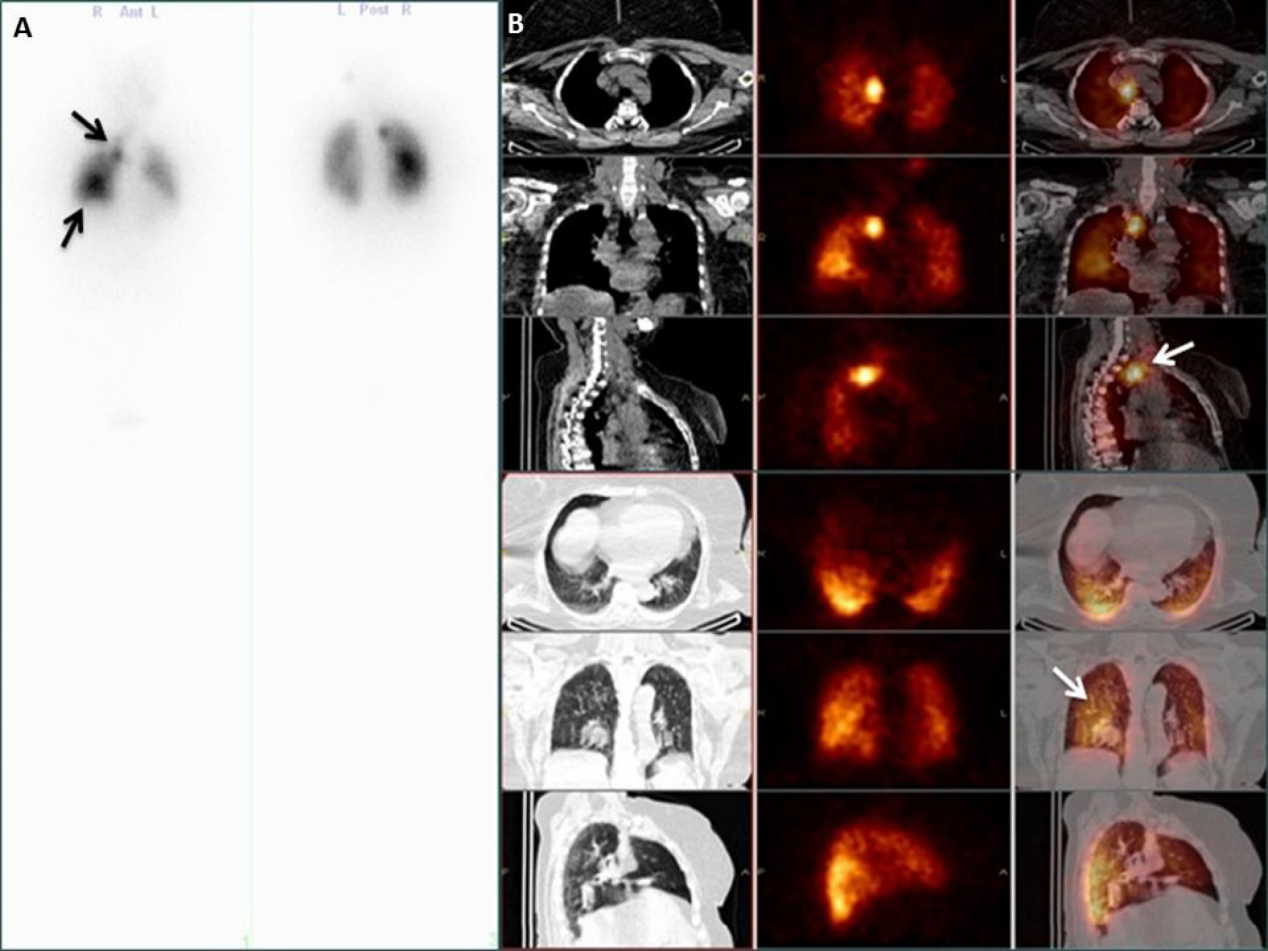
Anterior and posterior whole body scan (**A**) and SPECT/CT (**B**) images of a papillary cancer patient with lymph node and pulmonary metastases (arrows).

No evidence of tumor was detected by SPECT/CT in 78.3% of cases (Table 2). Local residual tumor was observed in 6 patients (1.8%), lymph node metastases were detected in 61 cases (18.8%), lung and bone metastases were found in 13 (4.0%) and 5 (1.5%) patients, respectively. In the ATA low risk category (*n* = 138), 91% of patients were tumor-free; lymph node, lung and bone metastases were detected in 10, 2 and 1 cases, respectively. In the ATA intermediate category (*n* = 159), no evidence of tumor was established in 75%. Lymph node, lung, bone and other metastases were diagnosed in 35, 3, 1 and 1 cases. Posttherapeutic SPECT/CT detected residual disease in every forth patient. ATA high risk patients (*n* = 26) were tumor-free only in 18%. Typical images of a patient with lymph node and pulmonary metastases are shown in Figure 1. Non-radioiodine avid lesions with suspected malignancy were detected in 8 cases (2.5%); these cases were further investigated by positron emission tomography/computed tomography (PET/CT), CT with contrast material or magnetic resonance imaging (MRI).

**Table 2:** The distribution of metastases according to SPECT/CT results in the original ATA risk categories (*n* = 323)

	ATA - low risk (138 patients)	ATA - intermediate risk (159 patients)	ATA - high risk (26 patients)	TOTAL
*No evidence of tumor*	125	122	6	***253***
*Lymph node metastases*	10	35	16	***61***
*Lung metastases*	2	3	8	***13***
*Bone metastases*	1	1	3	***5***
*Other metastases*	0	1	0	***1***

### Changes in risk classification and clinical stage based on SPECT/CT

The ATA risk stratification includes the WBS based RAI uptake outside the thyroid bed. In the present series, based on SPECT/CT results, patients with detectable residual disease were upgraded: the presence of lymph node metastases classified the patients to the intermediate risk, while incomplete tumor resection or distant metastases classified them to high risk of recurrence category. Patients without RAI uptake outside the thyroid bed previously categorized having intermediate or high risk were downgraded to low risk category except those with aggressive histology (Table 3A). Twenty patients were upgraded, while 95 patients downgraded, thus, the risk categories changed in 115 (35.6%) of cases. The risk distribution of the patients according to the ATA system before and after SPECT/CT differed significantly (*p* < 0.001), the Cohen’s *kappa* coefficient was 0.386, expressing a moderate agreement. The last ATA guideline does not recommend RAI ablation in the low risk category and the RAI therapy should be considered in the intermediate risk category. Without RAI treatment 103 (34.7%) patients would have been misclassified in the low and intermediate categories.

Table 3Changes in ATA risk classification and clinical stages based on SPECT/CT results

**Table:** A) ATA risk classification

		Before SPECT/CT			
		low	intermediate	high	TOTAL
*After SPECT/CT*	*Low*	124	83	5	212
	*Intermediate*	11	70	7	88
	*high*	3	6	14	23
	TOTAL	138	159	26	323

**Table:** B) Clinical stages

		Before SPECT/CT				
		I	II	III	IV	TOTAL
*After SPECT/CT*	*I*	208	0	0	0	208
	*II*	1	26	0	0	27
	*III*	3	0	31	0	34
	*IV*	7	2	5	40	54
	*TOTAL*	219	28	36	40	323

Changes in clinical staging were not so profound (Cohen’s *kappa*: 0.894), since the stage of young patients did not change even if they had lymph node metastases (Table 3B). However, 18 patients were upgraded, and 14 of them were classified to stage IV category, increasing the number of patients in stage IV by 25.9% (*p* < 0.001).

### Follow-up

Follow-up data were available in 315 cases; the median follow-up time was 37 months (range: 9–98 months). One patient died within one year and seven patients were lost for follow-up. Patients with confirmed residual tumor were treated by repeated surgery, RAI, irradiation or sorafenib in 23, 57, 9 and 6 cases, respectively, depending on the extension of the disease, type of tumor tissue and radioiodine resistance. Serum Tg, TgAb, neck ultrasound and other imaging modalities were used during long-term follow up. No evidence of tumor was found at 9–12 months after the RAI treatment in 251 (79.7%) cases. Incomplete biochemical response was detected in 20 cases (6.3%), residual tumor was evident in 44 patients (13.9%). Eighty-five percent of patients were tumor-free at the end of follow-up period. The incomplete biochemical response decreased to 2.5% (8 cases) while 12.1% (38 cases) of patients suffered from persistent thyroid cancer, seven of them died due to this disease.

### Comparison of the diagnostic value of the currently used risk stratification systems and SPECT/CT

Sensitivity, specificity, positive and negative predictive values and diagnostic accuracy of risk classification systems and SPECT/CT based on follow-up data at 9–12 months after radioiodine therapy are presented in Table 4A. All methods had acceptable sensitivity and NPV to predict the presence of DTC; however, the sensitivity of SPECT/CT compared to the ATA system was significantly lower (61% to 77%, *p* = 0.021). The ATA classification had the lowest specificity (47%) and diagnostic accuracy (53%) compared to the other systems tested (*p* < 0.001). The modification of ATA classification based on SPECT/CT findings significantly improved the specificity (73%) and diagnostic accuracy (72%) of this method (both *p* < 0.001). The results of SPECT/CT alone, without any other data, had the highest specificity (88%) and diagnostic accuracy (83%, *p* < 0.001).

Table 4Comparison of the diagnostic value of the currently used risk stratification systems and SPECT/CT

**Table:** A) At one-year after RAI treatment

	Sensitivity	Specificity	PPV	NPV	Diagnostic accuracy
***ATA***	76,6	47,4	27,1	88,8	53,3
***ETA***	70,3	62,2	32,1	89,1	63,8
***ATA after SPECT/CT***	65,6	73,3	38,5	89,3	71,7
***SPECT/CT***	60,9*	88,0**	***56,5***	***89,8***	82,5***

Positive predictive value (PPV), negative predictive value (NPV), Risk stratification of American Thyroid Association (ATA), Risk stratification of European Thyroid Association (ETA), Risk stratification of American Thyroid Association after SPECT/CT (ATA after SPECT/CT) and SPECT/CT alone (SPECT/CT).

*Sensitivity of SPECT/CT compared to the ATA classification was significantly lower (*p* = 0.021).

**Specificity of SPECT/CT was significantly higher than any other classification (*p* < 0.001).

***Diagnostic accuracy of SPECT/CT was significantly better than any other classification (*p* < 0.001).

**Table:** B) At the end of follow-up (median 37 months, *n* = 315)

	Sensitivity	Specificity	PPV	NPV	Diagnostic accuracy
***ATA***	80,4	46,5	20,4	93,3	51,4
***ETA***	73,9	60,6	24,3	93,1	62,5
***ATA after SPECT/CT***	78,3	72,9	33,0	95,1	73,7
***SPECT/CT***	71,7	86,6**	47,8	94,7	84,4***
***Risk at 1 year***	100*	93,3**	71,9	100	94,3***

*No significant differences in sensitivities were found except in case of one-year reclassification (*p* < 0.01).

**Specificities of the individual parameters differed significantly, the one-year reclassification had the highest value (*p* < 0.01). The specificity of SPECT/CT was also significantly better than the values of the ATA and ETA risk classifications (*p* < 0.001).

***Diagnostic accuracy of one-year reclassification was excellent but not significantly better than that of SPECT/CT (*p* = 0.59). Both method provided better prediction than ATA, ETA and ATA after SPECT/CT classifications (*p* < 0.01).

The usefulness of risk classification systems and SPECT/CT to predict the presence of thyroid cancer at the end of follow-up is shown on Table 4B. The reclassification of patients at one year was included in the analysis. No significant differences in sensitivities were found except in case of reclassification at one year, which was 100%. Specificity of the individual parameters differed significantly, the highest value was also found in case of one-year reclassification (93%, *p* < 0.01). Reclassification of patients at one year resulted in excellent diagnostic accuracy (94%). The specificity and the diagnostic accuracy of SPECT/CT alone were also high (87% and 84%), being significantly better (*p* < 0.01) than the values of the ATA and ETA risk stratification systems (ATA: 47% and 51%, ETA: 61% and 63%, respectively). The completion of ATA classification by SPECT/CT results provided better specificity (73%) and diagnostic accuracy (74%) than the ATA classification (*p* < 0.001). The diagnostic accuracy provided by the SPECT/CT to predict the presence or relapse of DTC at the end of follow-up was similar to the result of the one-year reclassification (*p* = 0.59). However, SPECT/CT results are obtained one year earlier. Diagnostic accuracies of different risk stratifications according to disease stages were also calculated (Table 5). The diagnostic accuracies of SPECT/CT at the end of follow-up in stage I, II, III and IV were 84.6%, 89.3%, 94.3% and 71.1%, respectively; these values were significantly higher than the diagnostic values of ATA and ETA risk stratifications in every stage.

**Table 5:** Comparison of the diagnostic accuracy of the currently used risk stratification systems, SPECT/CT and one-year data at the end of follow-up (median 37 months, *n* = 315) in different disease stages

	Stage I	Stage II	Stage III	Stage IV
***ATA risk***	57,5	50,0	22,9	44,7
***ETA risk***	71,5	82,1	11,4	44,7
***ATA after SPECT/CT***	75,2	67,9	74,3	68,4
***SPECT/CT***	***84,6***	***89,3***	***94,3***	***71,1***
***Risk at 1 year***	93,0	96,4	97,1	97,4

Risk stratification of American Thyroid Association (ATA risk), Risk stratification of European Thyroid Association (ETA risk), Risk stratification of American Thyroid Association after SPECT/CT (ATA after SPECT/CT) and SPECT/CT alone (SPECT/CT).

The role of SPECT/CT in predicting the disease outcome was further investigated by binary logistic regression analysis; age, TNM stage, clinical staging, histology, ATA, ETA risk classification and SPECT/CT were included to the model. The age, T, M stage and the SPECT/CT result proved to be the independent predictors of the outcome at one year. These determining factors were completed by ETA risk at the end of follow-up. SPECT/CT results were the strongest predictors in both models (*p* < 0.001).

## DISCUSSION

The postoperative management of DTC is based on the risk stratification of patients. However, different risk classification systems are used in the US, in Europe, and in other parts of the world [6, 7]. The risk classification mainly rests on the pathological results and surgical findings. The ATA risk classification contains the results of WBS after RAI; however, performing WBS is not obligatory. In the last few years several articles have been published evaluating the advantages of additional SPECT/CT over WBS alone in the management of DTC patients [11, 17]. Investigating 148 consecutive patients, SPECT/CT significantly reduced the number of equivocal findings on WBS and simultaneously was more accurate in the characterization of focal iodine accumulation in one fifth of patients [23]. The important diagnostic impact and the superiority of SPECT/CT over planar scintigraphy in cases of inconclusive lesions were also highlighted by others [24–30]. Despite of the obvious advantages of the hybrid imaging method, it is not a routine procedure in the world.

In this study, the role of SPECT/CT was evaluated in early risk classification of patients with DTC and in prediction of long-term prognosis compared to the risk of relapse determined by ATA and ETA risk classifications. To our best knowledge, so far our study has had the largest number of DTC patients with the longest follow-up time investigated by SPECT/CT. Moreover, this is the first study where the diagnostic value of combined imaging with additional SPECT/CT to predict the long-term outcome of DTC was compared to the usefulness of ATA and ETA risk stratifications.

Residual tumor was detected by post-radioiodine SPECT/CT in 22% of patients and this was unexpected in the majority of cases. The results of SPECT/CT basically modified the management in a considerable ratio of patients. The information about the lack of residual disease was equally important. The ratio of reclassified cases by SPECT/CT was high (36%). The majority of reclassifications moved the patients towards lower risk categories. This reclassification influences the treatment and follow-up e.g. the TSH target values and the frequency of follow-up visits. The detection of non-RAI avid lesions by SPECT/CT has also crucial importance as the loss of RAI accumulating capability means that this tumor will be resistant to RAI treatment and other treatment options are required e.g. irradiation or sorafenib treatment.

In prognostic models of disease outcome evaluated by binary logistic regression analysis, age, T, M stage and SPECT/CT results were found as independent predictors; The result of SPECT/CT was the strongest determining factor both at one-year evaluation and at the end of follow-up.

We tested two different applications of the post-radioiodine therapy SPECT/CT. Using the ATA risk categories, a large proportion of patients had to be reclassified based on the SPECT/CT results. Further, when post-radioiodine therapy SPECT/CT was used as the sole predictor of outcome, its specificity and diagnostic accuracy was significantly higher than any of the other currently used risk stratification systems. Using the SPECT/CT results alone, its sensitivity in predicting residual disease at one-year was lower than that of the ATA classification without SPECT/CT data; however, this difference disappeared by the end of follow-up. The lower sensitivity may be explained by the fact that very small metastatic foci are below the detection limit of SPECT/CT.

The response to the initial therapy is essential in determining long-term outcome. It has also been proven in our investigation that reclassification of patients at one-year based on the residual disease has the highest sensitivity, specificity and diagnostic accuracy predicting long-term outcome.

It is worth to mention that the ratio of follicular cancer (with potentially poor prognosis) was relatively high in our patients’ cohorts, probably due to marginal iodine deficiency in Hungary. The ratio of TgAb positive patients was also higher than expected [31, 32]. In TgAb positive cases, Tg cannot be used as a tumor marker for the follow-up. Therefore, the role of imaging methods in the TgAb positive patient population is even more important.

In our study, the residual disease was responsible for the biochemically or structurally incomplete response in the vast majority of patients and not a relapsing tumor was detected. It is possible that previous methods e.g. earlier Tg assays were not enough sensitive to detect the residual disease, however the follow-up time in this study is not enough long to withdraw final conclusion.

## CONCLUSIONS

In conclusion, SPECT/CT after radioiodine treatment is a useful tool in the early classification of DTC patients and largely influences treatment strategy. ATA and ETA risk classification systems are sensitive and have high negative predictive values, but are less specific when compared to post-radioiodine therapy SPECT/CT. Due to its better diagnostic accuracy, post-radioiodine therapy SPECT/CT can greatly facilitate staging, risk classification and management of DTC. We suggest that post-radioiodine therapy SPECT/CT should be included in the risk classification of patients with DTC.

### Informed consent

For this type of study formal consent is not required. The collection and analysis of patients’ data were approved by the Institutional Research Ethical Committees of both Universities.
